# Sjögren’s disease complicated by TAFRO syndrome: a case report of fever and lymphadenopathy with literature review

**DOI:** 10.3389/fmed.2026.1947691

**Published:** 2026-10-29

**Authors:** Yongmei Han, Xiayan Xu, Qianru Gu, Cheng Xu, Ning Shen, Xian Wang

**Affiliations:** 1 Department of Rheumatology, Sir Run Run Shaw Hospital, Zhejiang University School of Medicine, Hangzhou, Zhejiang, China; 2 Department of Pathology, Sir Run Run Shaw Hospital, School of Medicine, Zhejiang University, Hangzhou, Zhejiang, China; 3 Department of Medical Oncology, Sir Run Run Shaw Hospital, School of Medicine, Zhejiang University, Hangzhou, Zhejiang, China

**Keywords:** autoimmune disease, Castleman disease, rituximab, sjögren's disease, TAFRO syndrome

## Abstract

TAFRO syndrome, a fulminant subtype of idiopathic multicentric Castleman disease (iMCD), is characterized by thrombocytopenia, anasarca, fever, reticulin fibrosis, renal dysfunction, and organomegaly. Its occurrence in patients with pre-existing Sjögren's disease (SjD) is rare and poses significant diagnostic and therapeutic challenges. A 59-year-old male with a 10-year history of SjD complicated by primary biliary cholangitis (PBC) presented with a 3-month history of recurrent epistaxis, fever, and lymphadenopathy. Laboratory findings revealed severe thrombocytopenia, markedly elevated inflammatory markers, and positive anti-SSA/Ro and AMA-M2 antibodies. PET-CT demonstrated generalized lymphadenopathy, splenomegaly, and bilateral pleural effusions. An extensive workup excluded infection, lymphoma, and hemophagocytic lymphohistiocytosis (HLH). Excisional lymph node biopsy confirmed Castleman disease (plasmacytic type), and integration of clinical and pathological findings established the diagnosis of TAFRO syndrome. The patient failed to respond to high-dose corticosteroids, intravenous immunoglobulin, and dexamethasone combined with ruxolitinib. Definitive disease control was ultimately achieved with the RPAD chemotherapy regimen (rituximab, bortezomib, doxorubicin, and dexamethasone), leading to sustained clinical remission with normalized platelet count at 12-month follow-up. This case highlights that TAFRO syndrome can arise on the background of chronic autoimmune disease as a life-threatening cytokine storm, and may be mistaken for refractory HLH. Early recognition through systematic exclusion of mimics and histological confirmation is essential. In patients who fail conventional and cytokine-directed therapies, intensive multi-agent chemotherapy such as the RPAD regimen may be an effective salvage option.

## Introduction

TAFRO syndrome is an acute, fulminant cytokine storm disorder defined by the constellation of Thrombocytopenia, Anasarca, Fever, Reticulin fibrosis, Renal dysfunction, and Organomegaly (1). As the subtype of idiopathic multicentric Castleman disease (iMCD) with the poorest prognosis, it demands the most urgent therapeutic intervention; any delay in recognition can be life-threatening. First described by Takai et al. in 2010, the syndrome presents with complex and variable clinical features that frequently mimic autoimmune diseases, infections, and malignancies, thereby posing substantial diagnostic challenges (2). Herein, we report a patient with a 10-year history of Sjögren's disease (SjD) complicated by primary biliary cholangitis (PBC), who presented with fever, lymphadenopathy, and severe thrombocytopenia. Through a systematic diagnostic workup, a final diagnosis of TAFRO syndrome was established. This case aims to alert clinicians that TAFRO syndrome must be included in the differential diagnosis when patients with underlying autoimmune disease present with refractory systemic inflammatory manifestations.

## Case presentation

A 59-year-old male with a more than 10-year history of xerostomia and xerophthalmia, without a definite diagnosis or specific treatment, presented with a 3-month history of recurrent epistaxis, fever, and lymphadenopathy. Physical examination revealed multiple painless, moderately firm, enlarged lymph nodes in the cervical and supraclavicular regions. Laboratory investigations showed severe thrombocytopenia, markedly elevated erythrocyte sedimentation rate (ESR) and C-reactive protein (CRP), elevated immunoglobulin G (IgG), and significantly increased alkaline phosphatase (ALP) and gamma-glutamyl transferase (*γ*-GTP). Autoantibody testing was positive for antinuclear antibody (ANA), anti-SSA/Ro, anti-Ro-52, and anti-mitochondrial M2 (AMA-M2) antibodies. Serum cytokine profiling revealed a mildly elevated IL-6 level with VEGF within normal range. A decreased salivary flow rate and markedly reduced tear secretion on Schirmer's test confirmed a baseline diagnosis of Sjögren's disease (SjD) complicated by primary biliary cholangitis (PBC). There was no other relevant personal or family history. Disease course was shown in Figure 1.

**Figure 1:**
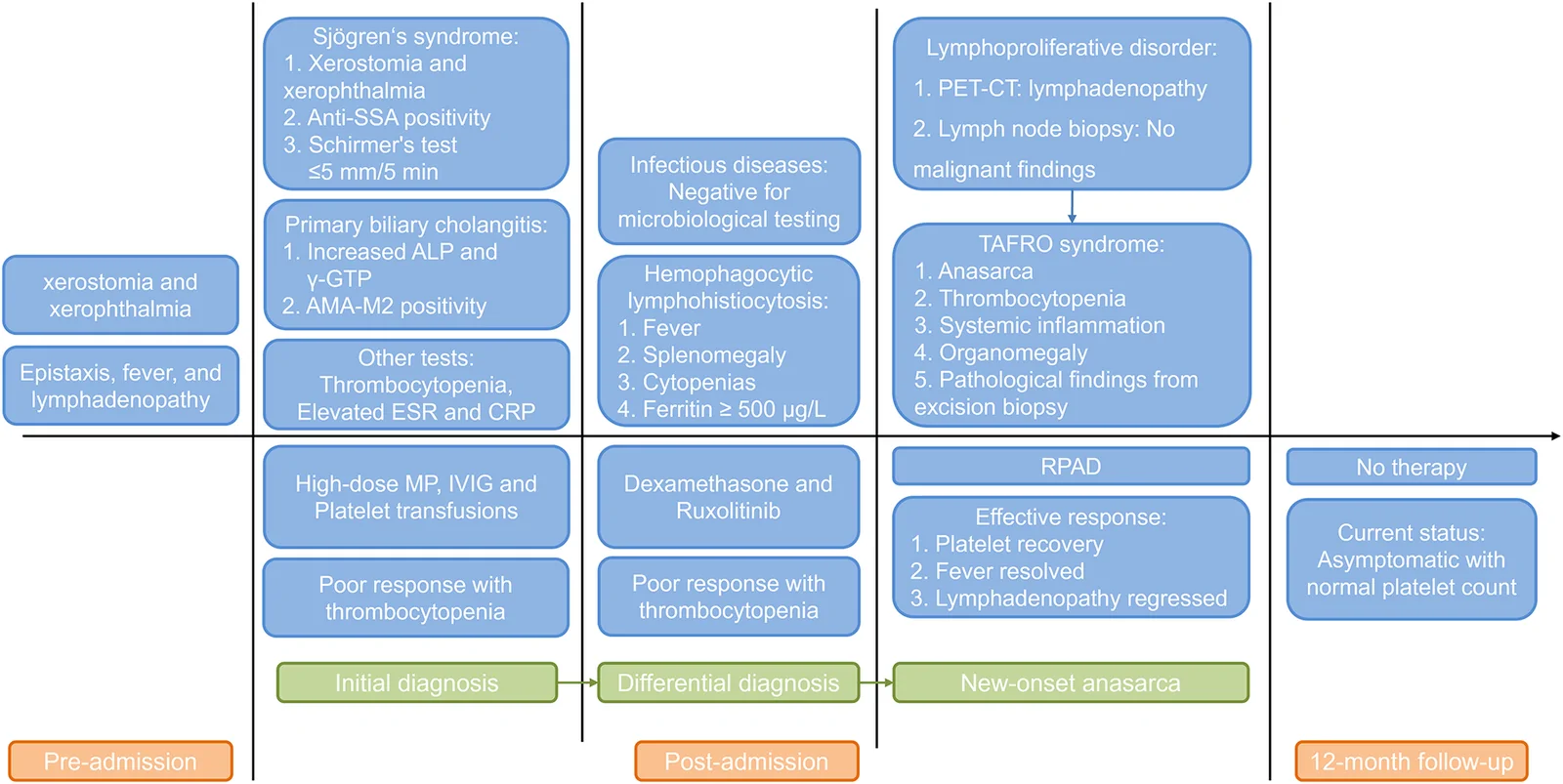
Graphical overview of disease course and therapeutic regimen. ALP, alkaline phosphatase; *γ*-GTP, gamma-glutamyl transferase; AMA-M2, anti-mitochondrial M2; ESR, erythrocyte sedimentation rate; CRP, C-reactive protein; MP, methylprednisolone; IVIG, intravenous immunoglobulin; RPAD, rituximab, bortezomib, doxorubicin, and dexamethasone.

A systematic differential diagnosis was undertaken. Infectious diseases were first excluded through extensive microbiological testing of serum, respiratory secretions, urine, and stool, as well as tuberculosis screening, all of which returned negative. Lymphoma and hemophagocytic lymphohistiocytosis (HLH) were then rigorously evaluated. Peripheral blood smear and serial monitoring of triglycerides, fibrinogen, and ferritin revealed no evidence of HLH, as well as slightly elevated sCD25 and mildly decreased NK activity. Bone marrow aspiration and biopsy showed no tumor cell infiltration or hemophagocytosis. PET-CT demonstrated generalized lymphadenopathy (involving the cervical, mediastinal, retroperitoneal, and inguinal regions), splenomegaly, and bilateral pleural effusions, a pattern highly suggestive of a lymphoproliferative disorder (Figure 2). An excisional biopsy of the most PET-avid superficial lymph node was subsequently performed. Histopathological examination revealed partial effacement of the nodal architecture, atrophic germinal centers, a diffuse interfollicular infiltrate of mature plasma cells, and proliferation of high endothelial venules. Immunohistochemistry confirmed polytypic light chain expression, with positive CD21 staining demonstrating an intact follicular dendritic cell meshwork, without abnormal overexpression of BCL2. These findings were consistent with Castleman disease, plasmacytic type (Figure 3).

**Figure 2:**
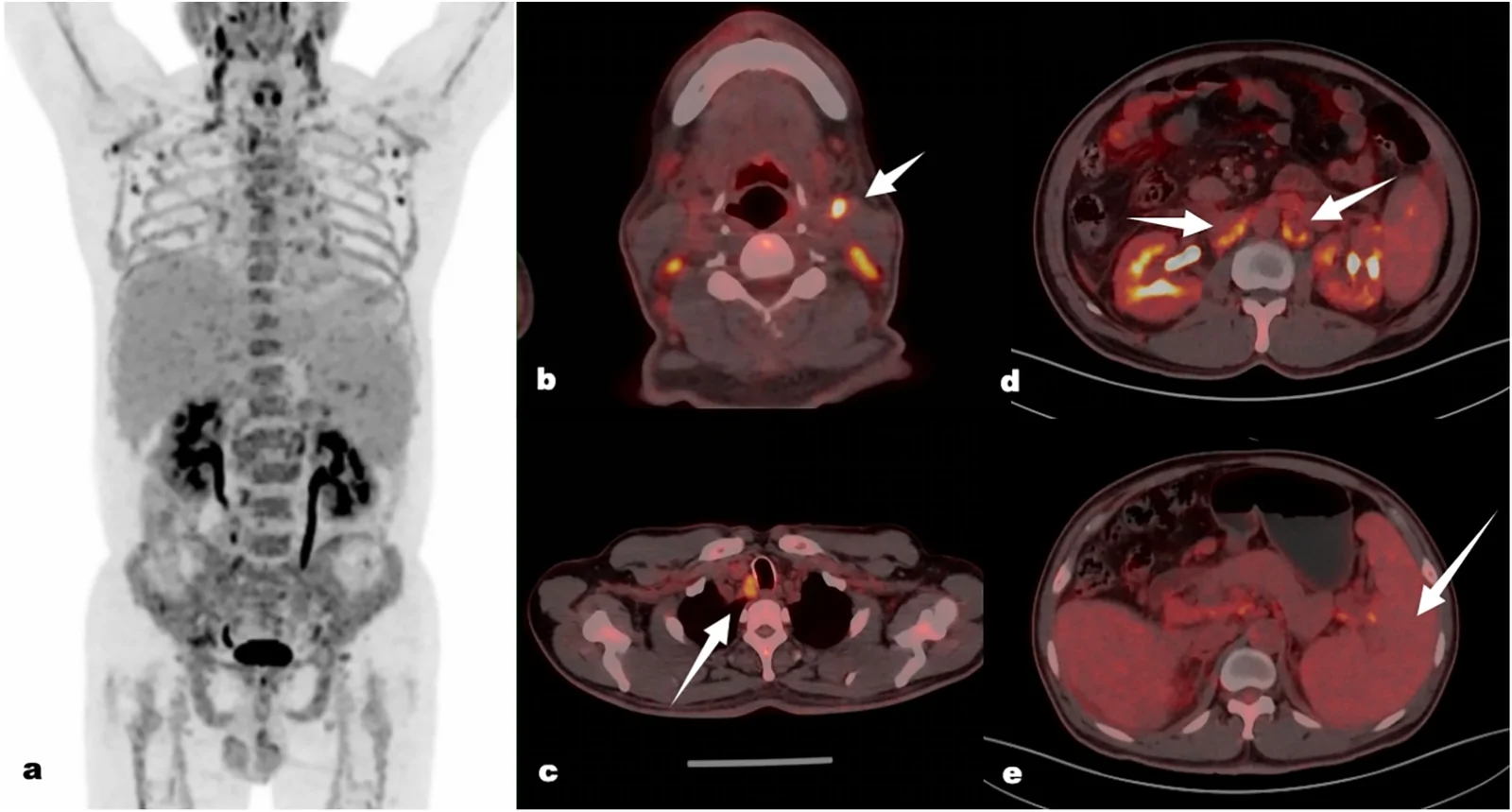
Positron emission tomography/computed tomography (PET-CT) findings. **(a)** Multiple FDG-avid lymph nodes are observed throughout the body, some of which are plump and enlarged in the cervical **(b)**, thoracic **(c)**, and abdominal **(d)** regions. **(e)** Splenomegaly with increased FDG uptake is also noted.

**Figure 3:**
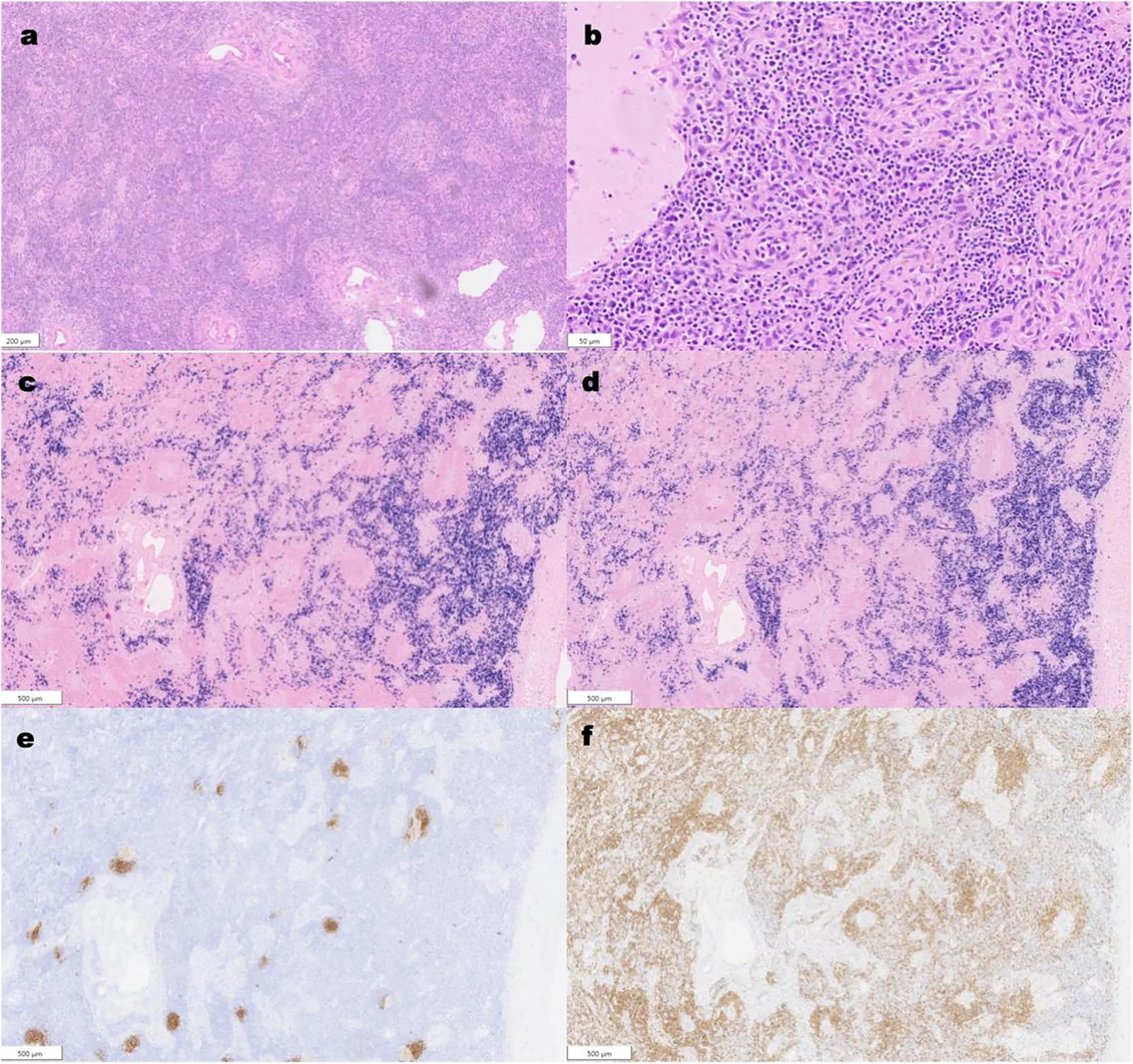
Histomorphological features by HE staining and immunohistochemical profiles. **(a,b)** HE staining shows partial effacement of the normal lymph node architecture, obliteration of the lymphatic sinuses, atrophy of the germinal centers in lymphoid follicles, and abundant diffuse infiltration of mature plasma cells in the interfollicular areas, with some cells extending into the subcapsular sinuses. Hyperplasia of high endothelial venules is observed in the interfollicular areas, and small vessels are seen penetrating the germinal centers. **(c,d)** *in situ* hybridization for immunoglobulin kappa and lambda light chains separately reveals a polyclonal pattern. **(e,f)** Immunohistochemistry shows that follicular dendritic networks are positive for CD21, while BCL2 expression is negative.

Integration of the patient's clinical manifestations—fever, progressive anasarca, severe thrombocytopenia, splenomegaly—with the lymph node pathology confirming Castleman disease established the final diagnosis of TAFRO syndrome. The clinical course was challenging. Initial therapy with high-dose methylprednisolone (2 mg/kg/day) and intravenous immunoglobulin (IVIG), supported by two platelet transfusions, failed to achieve clinical improvement. Given the refractory course, a presumptive diagnosis of secondary HLH was considered, and treatment was escalated to dexamethasone combined with ruxolitinib. Despite this adjustment, the patient's condition remained refractory. Definitive disease control was ultimately achieved with the RPAD chemotherapy regimen (rituximab, bortezomib, doxorubicin, and dexamethasone). Following this treatment, the platelet count rose above 40 × 10⁹/L, fever resolved, and lymphadenopathy regressed, indicating an effective therapeutic response. At the 12-month follow-up, the patient remained asymptomatic with normalization of platelet count and inflammatory markers, including CRP and ESR. Repeat serum cytokine testing showed that the IL-6 level remained mildly elevated, while VEGF was not reassessed due to the absence of clinical indication given its normal baseline value and the sustained clinical remission. Of note, the patient developed acute ST-segment elevation myocardial infarction (STEMI), which was successfully managed with percutaneous coronary intervention (PCI). Other treatment-related adverse events, including opportunistic infections, cytopenias, were observed.

## Discussion

We report a case of TAFRO syndrome arising in a 59-year-old male with long-standing Sjögren's disease (SjD) and primary biliary cholangitis (PBC). According to the 2022 Castleman Disease Collaborative Network (CDCN) international consensus definition for idiopathic multicentric Castleman disease (iMCD)-TAFRO (3), our patient met all mandatory criteria including thrombocytopenia, anasarca, fever, and organomegaly, exhibited a supportive feature of bone marrow reticulin fibrosis (MF grade 1), and had excisional lymph node biopsy consistent with Castleman disease (plasmacytic type). An extensive workup excluded infections and malignancies, as well as autoimmune diseases which could not account for the refractory clinical course. Accordingly, our patient was classified as definite iMCD-TAFRO, with a total disease severity score of 9, corresponding to severe disease according to the 2019 updated Japanese diagnostic criteria and disease severity classification for TAFRO syndrome (4). In addition, the prognostic scoring system for TAFRO syndrome (TS-PSS) was applied with a score of 0, indicating low risk and a favorable prognosis (5), which was consistent with clinical remission at the 12-month follow-up.

Although SjD is designated as an exclusion criterion for the diagnosis of TAFRO syndrome, this stipulation is not applicable to our patient. Conversely, this case exemplifies a rare but increasingly recognized clinical intersection (6–13) As summarized in Table 1, a growing number of similar co-occurrences have been documented, suggesting this phenomenon is unlikely to be coincidental. Instead, it may represent a distinct pathological trajectory wherein a background of chronic autoimmune B-cell activation evolves into an acute, life-threatening cytokine storm.

**Table 1:** Case report of patients with sjögren's disease complicated by TAFRO syndrome.

No.	Author (Year)	Age/Sex	Diagnostic Basis for SjD	TAFRO Features	Treatment	Outcome
1	Li et al. (2019)	36/F	ANA 1:160, anti-SSA (+)	PLT↓, anasarca, fever, BM fibrosis, splenomegaly, renal dysfunction, VEGF↑↑, IL-6 normal, LN: HV-CD	Siltuximab + CS ineffective→R-CVP	Effective
2	Suzuki et al. (2020)	52/F	anti-SSA/SSB (+), ↓salivation	PLT↓, anasarca (PE/ascites/edema), fever, splenomegaly, renal dysfunction, IL-6↑, VEGF↑	PSL + mPSL pulse→CsA + tolvaptan + eltrombopag	Effective
3	Masuda et al. (2025)	58/F	anti-SSA (+), labial biopsy (+), HTLV-1 (+)	PLT↓, anasarca, fever, splenomegaly, LAP, BM fibrosis, renal dysfunction, IL-6↑, VEGF↑	mPSL + TCZ	Effective
4	Watanabe et al. (2023)	60/M	anti-SSA/SSB (+), labial biopsy FS ≥ 1	PLT↓, anasarca, fever, renal failure (HD), BM fibrosis, IL-6↑, VEGF↑, LAP	mPSL/TCZ ineffective→RTX→belimumab	Effective
5	Carvalho et al. (2026)	37/F	anti-Ro52 (+), lacrimal biopsy: LES (FS > 1)	PLT↓, anasarca, fever, HSM, LAP, BM fibrosis (MF-1), renal failure (HD), CRP↑↑, ferritin↑↑	mPSL→PSL→TCZ	Effective
6	Fujimoto et al. (2017)	32/F	Labial biopsy at age 14; anti-SSA/SSB (+)	PLT↓, anasarca (PE/ascites/edema), fever, LN: mixed-CD, BM fibrosis (MF-2), HSM, proteinuria, renal dysfunction	PSL 30 mg/day	Complete remission
7	Uribe-Ruiz et al. (2026)	33/F	anti-Ro > 200 (+), labial biopsy FS = 1.5, Schirmer (+)	PLT↓, anasarca, fever, HSM, LAP (mixed-CD), BM fibrosis (grade 3), renal failure (HD), renal biopsy: TMA + MPGN	mPSL pulse→TCZ SC	Effective
8	Iwasaki et al. (2022)	61/F	History of SjD, anti-SSA (+)	PLT↓, anasarca (PE/ascites), fever, LN: CD, BM fibrosis, renal dysfunction, proteinuria, IL-6↑, VEGF↑, renal biopsy: TMA	PSL + CsA	Effective

ANA, antinuclear antibody; BM, bone marrow; CD, Castleman disease; CsA, cyclosporine A; CS, corticosteroids; FS, focus score; HD, hemodialysis; HSM, hepatosplenomegaly; HV-CD, hyaline vascular Castleman disease; LAP, lymphadenopathy; LES, lymphoepithelial sialadenitis; LN, lymph node; MF, myelofibrosis; mixed-CD, mixed-type Castleman disease; MPGN, membranoproliferative glomerulonephritis; mPSL, methylprednisolone; PE, pleural effusion; PLT, platelet; PSL, prednisolone; R-CVP, rituximab, cyclophosphamide, vincristine, prednisone; RTX, rituximab; SC, subcutaneous; SjD, Sjögren's disease; TCZ, tocilizumab; TMA, thrombotic microangiopathy; VEGF, vascular endothelial growth factor.

The precise mechanisms linking SjD to TAFRO syndrome remain incompletely defined, but several shared immunological pathways offer a compelling framework. A central hypothesis implicates aberrant activation of the type I interferon (IFN) signaling pathway. In SjD, anti-SSA/Ro antibody positivity—as observed in our patient—often reflects persistent type I IFN pathway activation. Mirroring this, patients with TAFRO syndrome exhibit significant upregulation of type I IFN-responsive genes, particularly in CD8+ T cells, NK cells, and monocytes (14). This shared signature suggests that SjD may provide a chronically activated immunological “soil”, primed by sustained type I IFN signaling, upon which an acute trigger could precipitate a TAFRO-like decompensation. Furthermore, hyperactivation of the mTOR signaling pathway serves as another plausible link, given its established elevation in both the salivary gland infiltrates of SjD and the hyperinflammatory milieu of TAFRO syndrome. Abnormal B-cell proliferation, epitomized by the polytypic plasmacytic infiltration found in our patient's lymph node, and the subsequent autoantibody and cytokine cascades constitute a pathological continuum bridging these two entities (15). However, the absence of substantial IL-6 or VEGF elevation, together with the unavailability of interferon-stimulated genes (ISGs) testing in our case, preclude robust support for this hypothesis. Future prospective investigations with serial cytokine and transcriptomic profiling are warranted to clarify the mechanistic interface between these two entities.

This case underscores critical clinical lessons regarding diagnosis and management. Our patient's initial presentation—featuring severe thrombocytopenia, progressive anasarca with bilateral pleural effusions, fever, and splenomegaly—in the context of pre-existing SjD and generalized lymphadenopathy, should immediately raise suspicion for TAFRO syndrome, the most aggressive subtype of iMCD. The diagnostic process compellingly illustrates the necessity of exclusion. The differential must be systematic: infection must be ruled out, as was done with comprehensive microbiological testing, and hematological mimics like hemophagocytic lymphohistiocytosis (HLH) and lymphoma must be excluded via rigorous hematological and pathological evaluation. Our patient's bone marrow biopsy was negative for HLH and neoplastic infiltration, and the lymph node biopsy excluded lymphoma, crucially revealing histopathology consistent with Castleman disease (plasmacytic type). Accurate differentiation is paramount as it dictates therapy: TAFRO syndrome carries the poorest prognosis among iMCD subtypes and demands immediate, aggressive intervention. Delayed diagnosis can be fatal.

RPAD regimen (rituximab, bortezomib, doxorubicin, and dexamethasone) is not listed as standard first-line therapy for iMCD-TAFRO in the international guidelines. However, our patient had an exceptionally complex clinical scenario of TAFRO syndrome occurring in the setting of underlying Sjögren's disease, with clinical suspicion for secondary hemophagocytic lymphohistiocytosis (HLH), showing an atypical cytokine profile with only mildly elevated IL-6, in contrast to the prominent IL-6 overproduction typically observed in classic iMCD. Notably, rituximab within RPAD confers potential dual-targeting effects by suppressing pathological B-cells responsible for both TAFRO and SjD, a therapeutic rationale supported by published case reports of SjD-associated TAFRO (9). Thus, after the failure of conventional therapies including high-dose corticosteroids, intravenous immunoglobulin, and dexamethasone- ruxolitinib with life-threatening thrombocytopenia and progressive anasarca, RPAD was administered as a salvage intensive combination chemotherapy. Meanwhile, this off-label salvage strategy has been reported in a small Chinese real-world retrospective cohort for heavily pretreated, organ-threatening iMCD-TAFRO (16). International guidelines also recognize bortezomib-containing multi-agent combinations as potential options for refractory iMCD (3, 17). The 2025 Chinese Castleman Disease Guidelines also allow earlier deployment of bortezomib-based combinations for patients with severe iMCD (18).

From a mechanistic standpoint, the RPAD regimen offers complementary therapeutic actions that directly target the pathogenic drivers of TAFRO syndrome, including elimination of pathological lymphocyte and plasma cell populations, suppression of pathogenic cytokine, and broad antiproliferative control. On the other hand, proteasome inhibitor-based therapy for TAFRO shares similar efficacy in Chinese and international practices, with notable differences in guideline positioning, preferred regimens and timing. The international CDCN framework does not designate bortezomib as a standard second-line therapy (3). Rather, it is mainly applied in intensive salvage combinations such as VDT-PACE-R (bortezomib, dexamethasone, thalidomide, Adriamycin, cyclophosphamide, etoposide, rituximab) (19, 20), RVD (rituximab, bortezomib, dexamethasone) (21) or RPAD (16) for heavily pretreated, organ-threatening TAFRO after failure of IL-6/IL-6R blockade, cyclosporine or rituximab monotherapy. By contrast, the 2025 Chinese Castleman guidelines recommend BCD regimen (bortezomib, cyclophosphamide, dexamethasone) as the preferred proteasome inhibitor-containing strategy for refractory or severe iMCD-TAFRO (18), which may be initiated earlier with life-threatening manifestations, partly due to the greater baseline disease severity requiring urgent cytoreductive intervention (22).

The sustained clinical remission at the 12-month follow-up, with normalized platelet count and inflammatory markers, as well as resolution of symptoms, confirms the efficacy of this intensive approach in carefully selected patients. However, the cardiac event merits careful interpretation. The timing of the event that occurred roughly six months after the last RPAD cycle, raises uncertainty regarding a direct regimen-related link, even though doxorubicin, bortezomib and rituximab are all associated with cardiac complications. Meanwhile, the patient harbored multiple conventional cardiovascular risk factors, including male sex, advanced age, and hypertension, which may have contributed substantially. Therefore, while causality with RPAD cannot be definitively ruled out, a multifactorial pathogenesis is most plausible where chemotherapy-related injury, the underlying pro-inflammatory state of TAFRO syndrome, and preexisting cardiovascular vulnerability collectively predisposed to this event. The favorable outcome following prompt PCI underscores the importance of close cardiac surveillance and prompt intervention in TAFRO patients receiving intensive multi-agent chemotherapy, particularly those with baseline cardiovascular risk.

## Conclusion

In conclusion, the co-occurrence of Sjögren's disease (SjD) and TAFRO syndrome likely signifies more than a chance association; it is a model of immunopathological progression from chronic autoimmunity to acute hyperinflammation. For the clinician, early recognition is the key determinant of survival. When a patient with SjD presents with unexplained fever, anasarca, and severe thrombocytopenia, TAFRO syndrome must enter the differential. This triggers the urgent, parallel workstreams of lymph node biopsy for histological confirmation and the exclusion of infectious and malignant mimics, allowing for the prompt initiation of intensive, often multi-modal immunosuppressive therapy.

## Data Availability

The original contributions presented in the study are included in the article/Supplementary Material, further inquiries can be directed to the corresponding author.
